# Sol-Gel Silica Nanoparticles in Medicine: A Natural Choice. Design, Synthesis and Products

**DOI:** 10.3390/molecules23082021

**Published:** 2018-08-13

**Authors:** M. Clara Gonçalves

**Affiliations:** 1 Departamento de Engenharia Química, Instituto Superior Técnico, Universidade de Lisboa, Av. Rovisco Pais, 1049-001 Lisboa, Portugal; clara.goncalves@tecnico.ulisboa.pt; Tel.: +351-8419934; 2 CQE, Centro de Química Estrutural, Instituto Superior Técnico, Universidade de Lisboa, 1049-001 Lisboa, Portugal

**Keywords:** sol-gel, silica, nanoparticles, Stöber, LaMer, reverse emulsion, biogeochemical cycle

## Abstract

Silica is one of the most abundant minerals in the Earth’s crust, and over time it has been introduced first into human life and later into engineering. Silica is present in the food chain and in the human body. As a biomaterial, silica is widely used in dentistry, orthopedics, and dermatology. Recently amorphous sol-gel SiO_2_ nanoparticles (NPs) have appeared as nanocarriers in a wide range of medical applications, namely in drug/gene target *delivery* and imaging *diagnosis*, where they stand out for their high biocompatibility, hydrophilicity, enormous flexibility for surface modification with a high payload capacity, and prolonged blood circulation time. The sol-gel process is an extremely versatile *bottom-up* methodology used in the synthesis of silica NPs, offering a great variety of chemical possibilities, such as high homogeneity and purity, along with full scale pH processing. By introducing organic functional groups or surfactants during the sol-gel process, *ORMOSIL* NPs or mesoporous NPs are produced. Colloidal route, biomimetic synthesis, solution route and template synthesis (the main sol-gel methods to produce monosized silica nanoparticles) are compared and discussed. This short review goes over some of the emerging approaches in the field of non-porous sol-gel silica NPs aiming at medical applications, centered on the syntheses processes used.

## 1. Introduction

Silica (SiO_2_) has been used throughout history in the manufacturing of glass, ceramics, concrete, mortar, sandstone and silicones [1]. Today, silica is present in an impressive variety of everyday life products, such as glass and tableware, vitroceramics, domestic and industrial water membranes, plastics, paper, paints, silicones, semiconductors, fiber and optical glasses, electronic, optoelectronic, aerospace, and defense products [2,3,4], to mention some of them. As regards healthcare and medicine, SiO_2_ is a current material in dentistry (tooth implants, ceramic pastes) [5,6], orthopedics (bone implants, scaffolds) [7,8], dermatology [9] and specialized medical devices (ophthalmological and bio-glasses, scaffolds) [10,11,12,13]. The coming of nanomedicine opened the door to new engineered materials/devices opportunities, colloidal silica having pride of place among micro and nanoparticles (NPs) [14,15,16,17,18,19,20,21,22,23,24].

Nanomedicine initiated a new attitude towards conventional medicine, where challenges are addressed using a *bottom-up* rather than *top-down* approach, medical actions are performed at a single cell level, tailor made therapeutic prescription are performed, *theranosis* (where diagnosis and therapeutic meet) is designed [25]. *Nanomedicine* is based on *nanocarriers* (nanosystems, nanoplatforms, NPs) capable of simultaneous targeting, sensing, signaling, and drug releasing distinct drugs/markers, with different pharmacokinetics/pharmacodynamics, to elaborate optimized *tailor-made* treatments/diagnosis. Cell tracking (in regenerative medicine or in the early detection of cancer) is carried out across all major imaging modalities—like magnetic resonance imaging (MRI), magnetic particle imaging (MPI), computed tomography (CT), positron emission tomography (PET), single-photon emission computed tomography (SPECT), to ultrasound (US), and optical (fluorescent) imaging [26] and journals like *Cancer Imaging* and *Cancer Imaging and Diagnosis*. Spatial and temporal controlled drug delivery (throughout injectable or oral nanocarriers) redesigned disease treatment ([27,28,29,30,31,32] and *Advanced Drug Delivery Reviews*). While introducing thousands of times less drug into the body nanomedicine scaled down the possibility of side effects, like tissue or organ inflammation or destruction, and at the same time increased the localization of pharmaceutical drugs in the diseased tissue. Moreover, engineered NPs maybe fine-tuned relatively to thermal, electrochemical, catalytic, electronic and magnetic properties or designed to respond to micro-environmental (pH sensitivity, enzyme sensitivity) or external (magnetic field, light) triggers [33,34,35,36,37].

**Figure 1:**
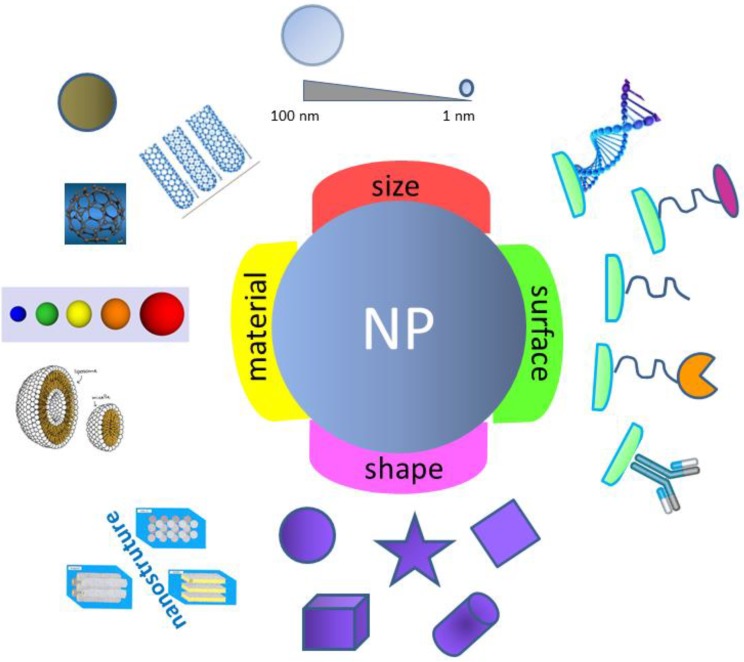
Engineered multifunctional NP.

As a nanocarrier, silica competes with biological materials (like phospholipids, lipids, lactic acid, sugars, proteins such as dextran or chitosan, virus), synthetic polymers (carbon or silicon-based), and other inorganic materials (ceramics, metals) (Table 1, Figure 1). Silica’s high thermal stability, chemical inertia (at PTN conditions), microbial attack immunity, along with strong surface energy, high hydrophilicity and biocompatibility, impermeability (or weak permeability), great chemical versatility (due to the possibility of in situ/ex situ physical functionalization, through second order chemical bonding, or chemical functionalization, in highly specific conditions), high loading capacity for drugs/chemicals/therapeutic molecules associated with ease of processing [38,39] are the properties that made it so special, a cost-effective, hard to beat material. Furthermore, amorphous silica and silicates are generally recognized as safe by the U.S. Food and Drug Administration [40], to be used as oral delivery ingredients in amounts up to 1500 mg per day, and have been widely applied as an additive in cosmetics, food, and oral drugs [41,42].

Sol Gel Gateway [43] lists the *on business* sol-gel companies, according to which CeraMem Corporation (Waltham, MA, USA), Quantum Dot Corporation (Hayward, CA, USA), General Engineering and Research (San Diego, CA, USA) and CD Creative Diagnostics (Shirley, NY, USA) stand out for their global market shares in sol-gel silica NPs. As to pharmaceutical and medical applications, the Canadian company PreveCeutical (Vancouver, BC, Canada) has developed (and patented) a sol-gel delivery platform for Nose-to-Brain Delivery of Therapeutic Compounds^®^, the first FDA approved CBD-based nose-to-brain delivery systems. Sol-gels are taken via nasal (systemic) administration and rapidly gel upon contact with mucosal tissue allowing a direct nose-to-brain delivery by slow releasing of drug/therapeutic molecules. By bypassing the stomach and intestines bioavailability may be improved (even when compared to nasal sprays and other newer delivery systems). Fluorescent ultra-small sol-gel SiO_2_ NPs (Cornell dots^®^) have emerged as a particularly fascinating fluorescent probe, which demonstrated excellent outcomes in the first human clinical trials (for cancer imaging) in patients with metastatic melanoma, being commercialized by Hybrid Silica Technologies (HST), a Cornell business startup (Ithaca, NY, USA) [44,45,46,47]. Magnetic (core-shell) sol-gel silica NPs (confering multifunctionality to the carrier) are particularly useful for externally magnetic guided systems in diagnosis, and hyperthermia treatments [48]. USA Cd Creative Diagnostics (Shirley, NY, USA) has commercialized a wide range of plain and core-shell (iron oxide magnetic core) sol-gel silica NPs while the French company NH TherAguix (Crolles, France) provides ultra-small sol-gel silica-based bismuth/gadolinium contrast agents. AuroLase^®^ Therapy (from Nanospectra Biosciences, Houston, TX, USA) utilizes *optically tunable* sol-gel nanoshells, able to convert light into heat and thermally destroy solid tumors without damaging adjacent healthy tissue. MRI/US Fusion Imaging and Biopsy in Combination with Nanoparticle Directed Focal Therapy for Ablation of Prostate Tissue^®^ and MR/US Fusion Guided Ultra-Focal Gold Nanoparticle Directed Photothermal Ablation of Prostate Gland Tumors^®^ are products in clinical trials. The Israeli (clinical-stage) company Sol Gel Technologies (Ness Ziona, Israel) focuses on developing and commercializing topical dermatological drug products, based on its proprietary sol-gel microencapsulation delivery system. Presently Vered^®^, Twin^®^ and Sirs-T^®^ are in phase II trials, while a generic candidate is in phase III (Table 2). In contrast to the limited number of sol-gel silica NPs currently under clinical tests a considerable number of sol-gel SiO_2_ NPs products/processes aiming medical applications are under investigation and some have been recently patented [49] (as proof of concept) and await clinical tests for national agency approval (Table 3).

Harkin Holdings (Tauranga, New Zeland) and B. Arkin Bio Ventures (Herzliya Pituach, Israel) are finance agencies that identify, invest in, and follow up innovative early and mid-stage companies directed to nanomedicine (with game-changing breakthroughs) in areas such as immunotherapy, cancer metabolism, microbiome, CNS, autoimmune diseases, orphan diseases and drug delivery nanoplatforms empowering this emerging I&D segment.

## 2. Silica Biogeochemical Cycle

Silica is the most abundant mineral in the Earth’s crust (~75 wt%). In the continental plate, silica’s prevailing geologic crystalline polymorphs are quartz, tridymite and cristobalite, (present in minerals such as feldspars, micas, zeolites or talcs), while the most common amorphous phases are obsidian (present in volcanic rocks like pozzolans, and ashes, and in rocks that suffered meteoric impact) and opals accounts for Gtons per year, vastly outweighing that produced industrially [50].

**Figure 2:**
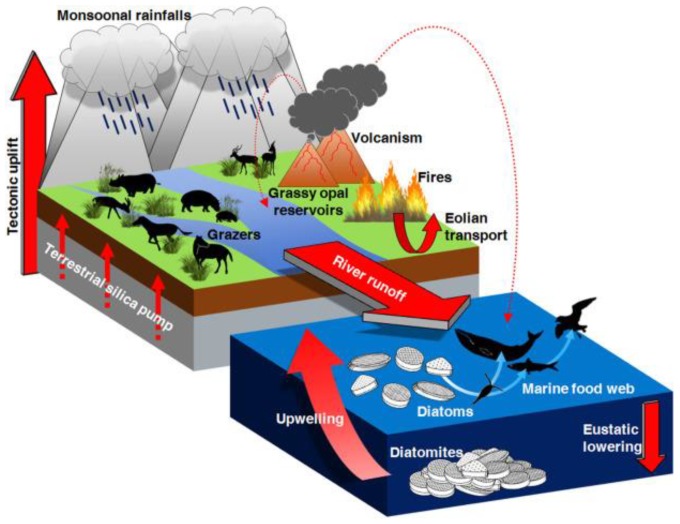
The diatomite deposition in the Mediterranean region. Reprint with permission from [51].

**Figure 3:**
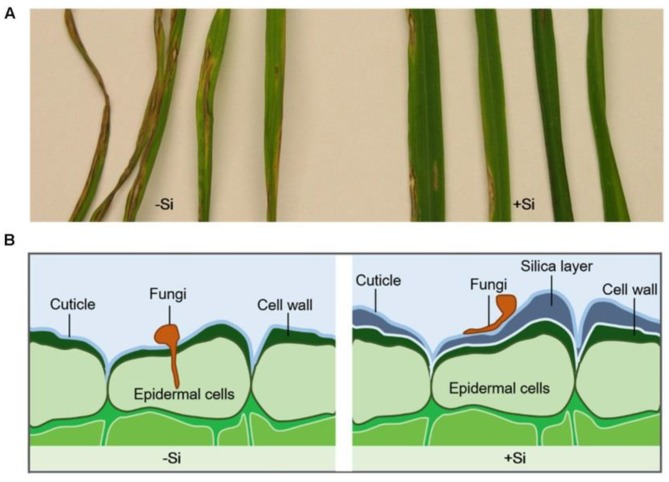
Leaf blast symptoms in rice after inoculated with *Magnaporthe grisea* for 10 days. Rice plants were continuously treated with (+Si) or without silicon (−Si) (**A**). Silica layer was formed in the cell wall of Si-treated plants and enhanced plant resistance to fungi infection by physical barriers (**B**). Reprint with permission from [57].

In the ocean plates, diatomaceous earth constitutes a significant amorphous silica (a-SiO_2_) source. This highly reactive biogenic silica deposit layer resulted from the silica-based skeletal remains of tiny aquatic organisms (diatomaceous and silicified phytoplankton) deposited over millions of years, and may include minor amounts of quartz, oxides of Ca, Mg, Fe and Al, and even some organic matter, depending on their location (Figure 2). More importantly, silica performs an essential role in many, if not all, forms of life [38]. The weathering and deterioration of rocks allows for high silica contend in soils (from <1 to 45% dry weight [52]) in the form of soluble [Si(OH)_4_] or Si(OH)_3_O^−^ species (with concentrations ranging from 0.1 to 0.6 mM [53]), allowing its uptake by plants, thus entering the food chain [54,55]. Although not traditionally thought of as an essential element in plants’ life cycles, Si is found in plants at concentrations varying from 0·1 to 10% (~10^3^–10^5^ mg kg^−1^, dry weight basis), an amount equivalent to, or even exceeding, several macronutrients [53]. Biogenic silica occurs in many single-cell organisms, as natural amorphous structures with the composition SiO*_n_*(OH)_4−2*n*_. The skeleta of diatoms (*Diatomeae*, 5 µm up to 60 µm) are formed with silica. Upper plants like *Equisetaceae* may accumulate 1 up to 10% silica (in dry weight), *poaceae*, *Equisetaceae* and *Cyperaceae* families have silica values >4% wt, the *Cucurbitales*, *Urticales* and *Commelinaceae* families accumulate an intermediate Si content (2–4% wt Si), while most other species contain trace amounts of silica. Nevertheless, silica is known to favor the healthy growth, development and reproduction of plants, increase their resistance to fungi, enhance their mechanical resistance, apart from not being detrimental when excessively collected [56] (Figure 3).

In the human body, silica’s effects in health, aging and disease are still being explored, notwithstanding its average content of ca. 260 ppm, very close to the magnesium level (accounting for 18 g in a person with 70 kg) [58,59]. Silica is pivotal in calcium phosphate nucleation, determinant in bone mineralization, growth and self-healing during dislocations and fractures [59]. Aluminum absorption and (eventual) intoxication in human body (particularly important in Alzheimer’s disease) may be prevented by silica ingestion, through the bonding to Al in the gastrointestinal tract [60,61]. Maintenance of health in the immune system and reduction of the risk of atherosclerosis are other of silica attributes [62]. Silica also accounts for the maintenance of tissue integrity by stabilizing the glycoproteins associated with collagen, thus playing a central role in the structural integrity of nails, hair, and skin [63]. Amorphous silica could enter in the human body via the gastro-intestinal tract (in colloidal form) and has been part of human dietary either as medical clay, or as food additive (labelled E551) [64,65]. Contrary to crystalline silica, associated with lungs cancer and silicosis [66,67], a-SiO_2_ NPs seem essentially nontoxic [68]. Any (eventual) inflammation process associated with a-SiO_2_ NPs is dependent on the NP size, shape and synthesis methodology, being no unambiguous linking of physical-chemical properties of a-SiO_2_ and human toxicity, bioavailability, yet possible [68,69,70,71,72,73,74,75,76].

## 3. Silica NP Design

Pharmaceutical design is currently based on the Quality by Design (QbD) concept, a new, systematic, risk-based methodology. QbD begins with predefined objectives and dwells on product and process understanding, along with process control [77,78,79,80]. QbD requires: (i) knowledge of the physiologic barriers NPs face within the human body, (ii) complete characterization of NPs materials, and (iii) fully understanding of the NP synthesis process.

### 3.1. NPs Physiologic Barriers

The biological performance (pharmacokinetics profiles, biodistribution, target recognition, therapeutic efficacy, inflammatory reactions and toxicity) of intravenously injected NPs is controlled by a complex array of interrelated physicochemical and biological factors, starting with *opsonization*, followed by *phagocyte* ingestion and ending with NPs *clearance*. [81,82,83,84]. Rapid blood clearance limits drugs/gene/therapeutic molecules/markers accumulation at target delivery sites, while NPs accumulation in macrophages (within clearance organs) initiates inflammatory responses, inducing toxicity [85].

Opsonization is the process by which a foreign organism or particle becomes covered with biologic proteins (opsonins), forming a coating (named *corona* by materials scientists or *opsonins* in pharmaceutics) thereby making it more visible to phagocytic cells. The exact mechanism through which opsonization is activated is complex and is not yet fully understood. When the opsonin proteins (blood serum components like laminin, fibronectin, C-reactive protein, type I collagen, components of the complement proteins such as C3, C4, and C5 and immunoglobulins [81,82]) come into close contact with engineered NPs, typically by random Brownian motion, they may adsorb on NPs surface through van der Walls, electrostatic, ionic or hydrophobic/hydrophilic attractive forces. Protein opsonization usually takes place in the blood circulation system and may hold from few seconds to many days to complete.

After opsonization, phagocytosis occurs. Macrophages (typically Kupffer cells of the liver or spleen) cannot directly identify the NPs themselves, but rather recognize (through specific, non-specific receptors or complement activation) opsonin proteins adsorbed to the NPs surface [81]. Macrophages may uptake foreign materials within a matter of minutes (after opsonization), increasing the phagocytosis rate for positively charged and bacteria-specific proteins and render them ineffective as nanocarriers [83].

Finally clearance occurs. The phagocytes begin to secret enzymes and other oxidative molecules (like superoxides, oxyhalide molecules, nitric oxide, and hydrogen peroxide) to ingest (chemically break down) the phagocytosed material [81]. Unfortunately, most non-biodegradable NPs cannot be degraded significantly by this process and, depending on their relative size and molecular weight will either be removed by the renal system or sequestered and stored in one of the mononuclear phagocyte system (MPS) organs.

**Figure 4:**
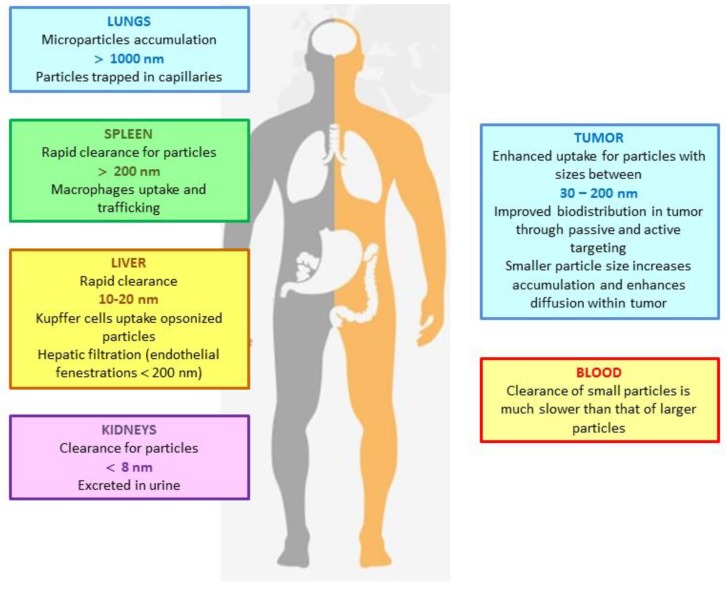
Major organ sites of NPs localization.

Renal clearance (based on physical filtration, dialysis) is a second optimal method for expelling NPs from the body. As a first approximation, removal by the renal system occurs only for polymeric molecules with a molecular weight of around 5000 or less, or inorganic NPs with hydrodynamic diameters smaller than 8 nm. Dendrimers (with molecular weights as high as 100,000) and NPs larger than 8 nm (if somehow broken down into fragments smaller than 6 nm after drug release) may also be cleared by the renal system [86]. Non-biodegradable NPs and degradation molecules with a molecular weight higher than the renal threshold typically become sequestered in the MPS organs (Figure 4).

Several methods have been developed to mask or camouflage NPs from the MPS or renal clearance. The most preferred of these methods is the adsorption or grafting of poly (ethylene glycol) (PEG) to the surface of NPs. Addition of PEG and PEG-containing copolymers to the surface of NPs resulted in an increase in the blood circulation half-life of the NPs by several orders of magnitude. This method created a hydrophilic protective layer around the NPs that is able to repel the absorption of opsonin proteins via steric hindrance, thereby blocking and delaying the first step in the opsonization process [81]. Moreover, PEGylation prevents NPs aggregation in solution, which helps keep them from forming a cluster once in blood vessels, where they could otherwise embolize and occlude blood flow resulting in microinfarctions at distant sites and organs.

As to toxicity, amorphous silica solubility (~120–170 ppm, at the temperature and pH of the body fluids, 36–37 °C and 7.35–7.45, respectively), allowed an easier silica elimination (as silicic or poly(silicic) acid) which are non-toxic and diffuse through the blood stream or the lymphatic system to be eventually cleared in the urine, preventing its accumulation in kidneys, liver or spleen (contrary to the crystalline polymorphs counterparts) [87,88]. Amorphous silica phases lacked the regular long-range order purposed by the classical crystal growth and dissolution models which difficult the understanding of its dissolution mechanism. Yet, a-SiO_2_ phases shared with the silica crystalline polymorphs the fundamental unit, the (SiO_4_)^4−^ tetrahedron, and short range structural order (at length scales up to 20 Å), despite variations in Si-O-Si bond lengths and angles. The a-SiO_2_ atomic scale disorder enabled the loss of surface (SiO_4_)^4−^ Q^3^ units into solution (creating vacancy islands) and keeping unchanged the a-SiO_2_ surface Gibbs energy. As a consequence, dissolution rates of amorphous silica phases, which involves an equilibrium between the solid phase and dissolve monomer Si(OH)_4_, scaled linearly with increasing driving force (undersaturation). At pH values above 8, the presence of [H_3_SiO_4_] ion in addition to Si(OH)_4_ is responsible for the high silica solubility at this pH values (as the concentration of Si(OH)_4_ in equilibrium with the solid silica phase is not pH dependent).

The US National Cancer Institute has pointed out that most engineered NPs are far less toxic than household cleaning products, insecticides used on family pets, or over-the-counter dandruff remedies, which are present at order-of-magnitude higher levels than the engineered NPs [89]. Moreover, in their use as carriers of chemotherapeutics in cancer treatment, engineered NPs are much less toxic than the drugs they carry.

### 3.2. NPs Characterization

NPs size and morphology, chemical and surface composition, along with crystallinity (or lack of) were used to explain limitations in clinical translation, NPs clearance and inflammatory processes [81,82,83,84,88].

As far as size is concerned, NPs may exhibit different size profiles and different shell thicknesses (in core-shell structures), showing in all cases an outstanding surface-to-volume ratio, responsible for an extremely active/reactive surface performance. Size determines in vivo distribution, intracellular uptake, toxicity, and targeting ability, influencing drug loading, drug release, and in vivo and in vitro stability. Smaller particles have a great risk of aggregation during storage and incubation in vitro, but have higher mobility and longer circulation half-life in vivo. To run an effective and reproducible biomedical NPs system, monosized distribution is required, usually between 10 and less than 200 nm.

As to shape, NPs may exhibit an extensive range of geometries—from spherical to tubular, through centric, eccentric and star like. While spherical NPs are good candidates for drug delivery, anisotropic structures can sometimes provide higher efficiencies in drug deliver (due to a more favorable configuration with the cell), although the sharp edges and corners may induce injuries to blood vessels. NPs may be hollow, dense, nanostructured, or in core-shell (eventually with multiple cores) structures, enhancing the NP load capacity and specific targeting ability.

NPs may differ in surface chemical composition, a critical parameter in determining their drug-loading efficiency, releasing profile, circulation half-life, tumor targeting and clearance from the body. A hydrophilic surface makes the NPs more resistant to the plasma proteins adsorption (preventing the formation of corona) and thus avoiding their recognition and uptake by the MPS. Coating the NP surface with a hydrophilic polymer (like PEG) or directly synthesizing NPs with hydrophilic surface compounds (in situ synthesis) are two strategies to overcome the challenge. Rapid opsonization and clearance is observed for NPs with excess positive surface charges [90].

As regards the renal system, neutral surface charge gives the highest chance to pass through renal filtration (and being excreted in urine), while both positively and negatively charged NPs adsorbed more serum proteins, increasing their hydrodynamic diameter and thus reducing their ability to be eliminated [91]. Unlike the long time clearance process taken by bile (or other MPS organs), the renal system removes the NPs from the body through the urine, with minimal side effects [88].

### 3.3. NPs Synthesis Methodologies

During the last decades, remarkable efforts have been made to develop novel NPs synthesis methodologies. Today, it is generally accepted that nanosize cannot be efficiently achieved by the traditional *top-down* methodologies (such as ball milling and lithography) but rather by *bottom-up* techniques. A *bottom-up* strategy looks faster, precise, and cost-effective.

A *bottom-up* strategy holds a large number of techniques (flame spray pyrolysis, chemical vapor deposition, and wet-chemistry methodologies like co-precipitation, hydrothermal, solvothermal and sol-gel) but research has been focused on sol-gel, as the synthesis is straightforward, scalable, easily controllable, time and energy saving. The sol-gel chemistry comprises chemical reactions involving colloidal particles in a sol, or between alkoxide-precursors and water, in a solution, leading to a highly porous amorphous gel product, in which a liquid phase (solvent, catalyst and eventually excess reactants) may be retained in bulks (3D), films (2D), fibers (1D), powders, and NPs (0D) products [39,92]. The sol-gel SiO_2_ NPs synthesis comprises four common methods: (i) colloidal routes, (ii) biomimetic syntheses, (iii) solution routes (base- and acid catalyzed)) and (iv) templated syntheses (the last one dedicated to mesoporous silica NPs, a topic outside the scope of this short review).

#### 3.3.1. Colloidal Route

**Figure 5:**
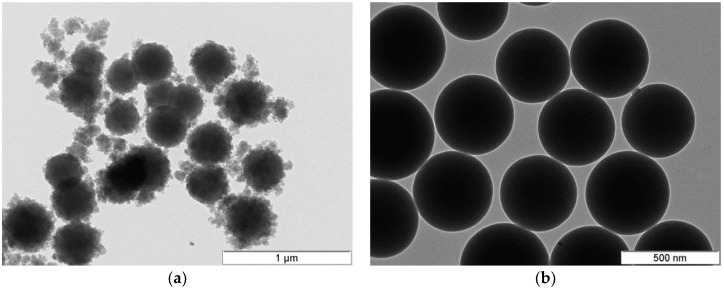
Sol-gel silica NPs growth through Ostwald ripening: (**a**) initial and (**b**) final stages.

In colloidal routes, sol-gel SiO_2_ NPs are formed in an aqueous medium through the supersaturation, polymerization and (eventual) precipitation of silica polymorphs. In the geological world silica NPs of ~1 nm (basically orthosilicic acid, a weakly acidic molecule with pK_a_ ~9.8) undergo rapid growth to 2–4 nm, at pH 2–3 (as pH_PZC_ ~2.2; ζ ~0 mV at pH ~2–3, facilitating NPs growth). As silica solubility increases well above pH 7 particles grow up to 4–6 µm by coalescence and Ostwald ripening (pH >> pH_PZC_, and ζ < −30 mV). At pH > 9 (the ionized form of monomeric silicic acid Si(OH)_3_O^−^ predominates) silica NPs cement to form a bulk gel, originating opal-like structures [38,39]. Ostwald ripening of silica particles originates from surface instability of silicon dioxide and is driven by differences in chemical potential between particles of different size and shape. The local radius of curvature and ratio of surface area to volume accounts for the particle’s surface energy, which is greater in the case of small particles or those with rough surfaces. Under kinetically favorable conditions these high surface energy particles dissolve preferentially, with the material being deposited onto particles with the largest radius. Silica dissolution proceeds via cleavage of siloxane bonds on the NPs’ surface (which is faster in amorphous structures), resulting in the release of soluble silicic acid (Figure 5).

Commercial precipitated silica, formed from sodium silicate solution and sulfuric acid, has the largest share of global market of silica particles (in classical industries), a position that is expected to grow in the next decade [2,3,4].

In the biological world plants, diatoms and sponges are capable of accumulating, storing and processing Si to create biogenic silica (at mild ambient conditions and under-saturated aqueous solutions of silicic acid). Several factors affected the process of natural silica condensation, namely concentration of silicic acid, temperature, pH, and the concentration of co-precipitating/nucleating agents (external small molecules and polymers) [93]. Plants started by taking up Si in the form of Si(OH)_4_] or Si(OH)_3_O^−^ (present in soils at concentrations as low as few mg kg^−1^). When the silicic acid concentration is in excess of 100–200 mg kg^−1^, polycondensation reactions occur at final location, forming silica polymers equal or higher in size than the critical nuclei size. The viable *nuclei* grow to form spherical NPs, as the absence of crystallographic patterns promotes isotropic spherical growth. The final SiO_2_ NPs are amorphous at the 1-nm length scale [94], built up from SiO_4_ tetrahedron with variable Si-O-Si angles and Si-O bond distances. However, a great variety of medium/long range order patterns may be found in nature (branched chains, structural motifs or even hierarchical patterned structures) resulting in different density, hardness, solubility, viscosity and composition values [54,55,93]. As the silica NPs reach 1–3 nm in size, they interact with plant cell walls (due to the negatively charged silica NPs surfaces, at neutral pH).

**Figure 6:**
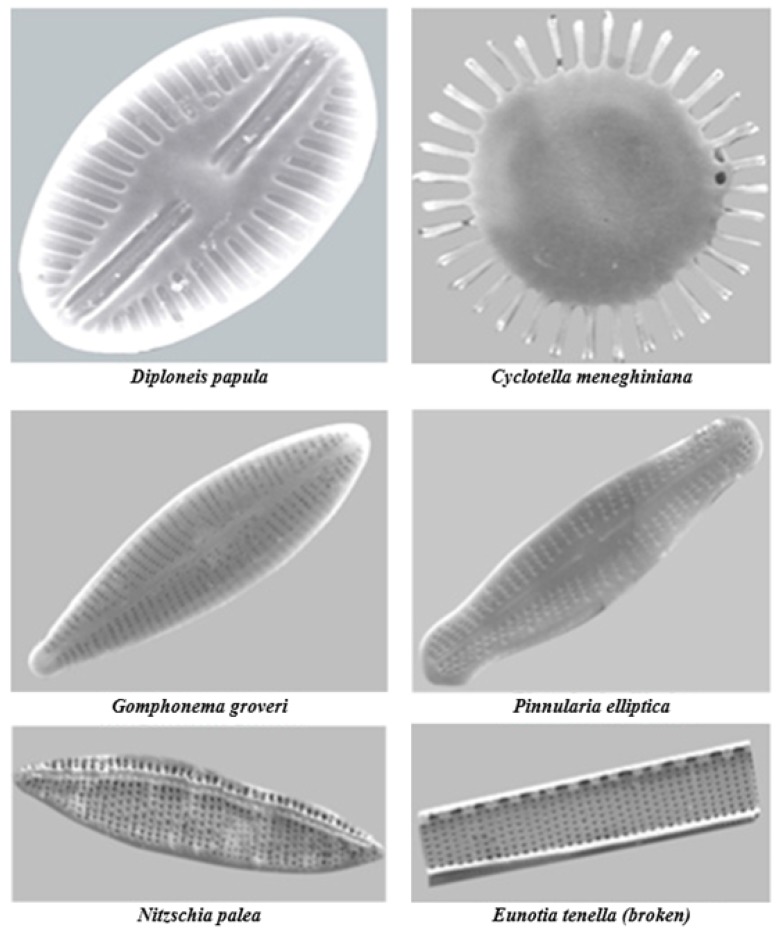
Photomicrographs of diatoms through Scanning Electron Microscope. Reproduce with permission from [101].

As to diatoms, there are more than 105 species with unique frustule architectures (Figure 6). The micro- and nano-sized diatoms can be also produced by cultivation, and here purification and chemical modification protocols are well established to generate pure active biohybrid materials [95]. Furthermore, the production of diatoms is environmentally friendly (compared to synthetic silica-based NPs), due to absence of toxic waste products and low energy consumption. Diatoms are considered to be harmless thanks to the amorphous silica structure [96], and food grade diatomaceous earth has been approved in the USA to feed animals and there are already several human grade diatomite silica microparticles products on the market in Europe and Australia [97]. The potential of silica diatoms for oral drug delivery applications, in intestinal (pH 7.2) and simulated GIT (pH 1.2–7.4) fluids [98,99,100] was recently demonstrated (Table 4).

#### 3.3.2. Biomimetic SiO_2_

Mimetic natural SiO_2_ production is gaining ground, and represents a source of inspiration for green eco-production processes. In biomimetic silica synthesis particle formation can occur by the use of certain co-precipitating/nucleating (biologic or biomimetic) agents, under neutral or acidic conditions. As research on the biogenic silica production has progressed, key molecules (such as silicateins, silaffin R5, proteins, peptides, carbohydrates, lipids, metal ions and phenolic compounds) that participate in the silicification of microorganisms have been found. Several studies have identified alternate amine-molecules as candidates for inducing silica precipitation from precursor compounds in vitro. These amine groups thus impart the silica with a strong positive surface charge (populated with -NH_3_^+^ groups, ζ ≥ 30 mV) in acid and neutral pH, thus stabilizing the silica sol and allowing the NPs growth through Ostwald ripening [113,114]. Spherical porphyrin-functionalized SiO_2_ NPs were biomimetically synthesized with diameters between 50 nm and 800 nm. However, high quality silica NPs with a diameter less than 50 nm still remains a long term challenge [115,116,117,118]. Nearly monodisperse SiO_2_ NPs, with tunable size between 10 nm and 200 nm, were synthesized in aqueous media by using lysine [119,120] and arginine [121,122] as base catalysis. Cationic block copolymer micelles [123] and cationic poly(acrylamine-co-2-(dimethylamine) ethyl methacrylate, methyl chloride quaternized) (poly (AM-co-DMC)) [124] and polyalylamine hydrochloride (PAH) [125] were used as colloidal template for the biomimetic deposition of 35 nm silica NPs. Protein immobilization (biomolecules encapsulation) within biomimetic silica NPs has been investigated for a wide variety of enzymes [126,127,128,129,130], bovine serum albumin (BSA) protein [131] and has even proved successful for the entrapment of different enzymatic proteins [132].

#### 3.3.3. Solution Route

**Figure 7:**
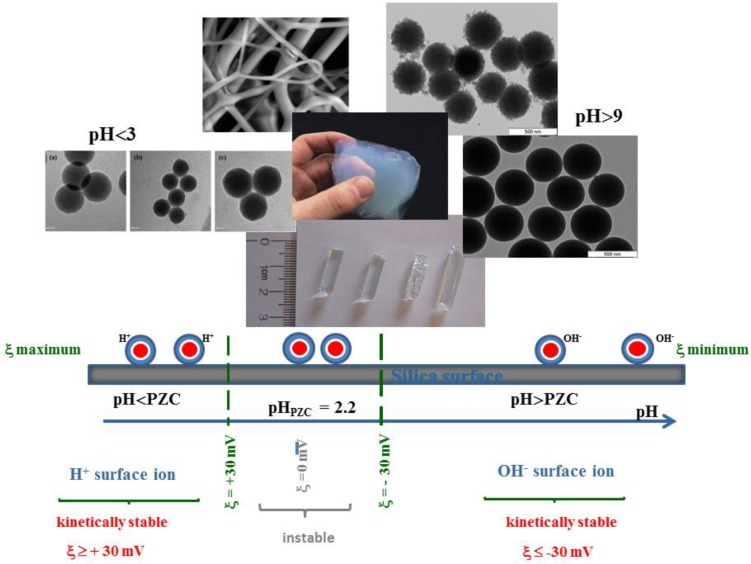
Sol-gel products diversity: the right choice of catalyst, pH value, water to silica precursor’s ratio, type of solvent and solvent to water ratio, type of silicon precursor, presence of chelating agent, and temperature, allow the control of SiO_2_ topology.

The *solution route* is the most common sol-gel synthesis process. Here metallic salts, metal alkoxides, or other organometallic precursors undergo hydrolysis and condensation, to form a wide range of sol-gel products. The right choice of catalyst, pH, water to silica precursor’s ratio (to control hydrolysis rate), type of solvent and solvent to water ratio (to enhance reactants mixing), type of silicon precursor (as R may have inductive and steric effects on hydrolysis rate), the presence of chelating agent (to control the relative hydrolysis to condensation rate) and finally the temperature, allow the control of SiO_2_ structure, size and/or morphology (Figure 7). Due to the hydrophobic nature of the alkyl groups organometallic precursors and water are not miscible, and the addition of a common solvent (usually an alcohol) becomes mandatory to promote miscibility between reactants. In the case of silica synthesis, the low polarity of the Si-O bond in silicon alkoxide (the Si atom bear δ^+^ = 0.32 low positive charge in TEOS) is responsible for the slow sol-gel progress, rendering catalysis essential.

**Figure 8:**
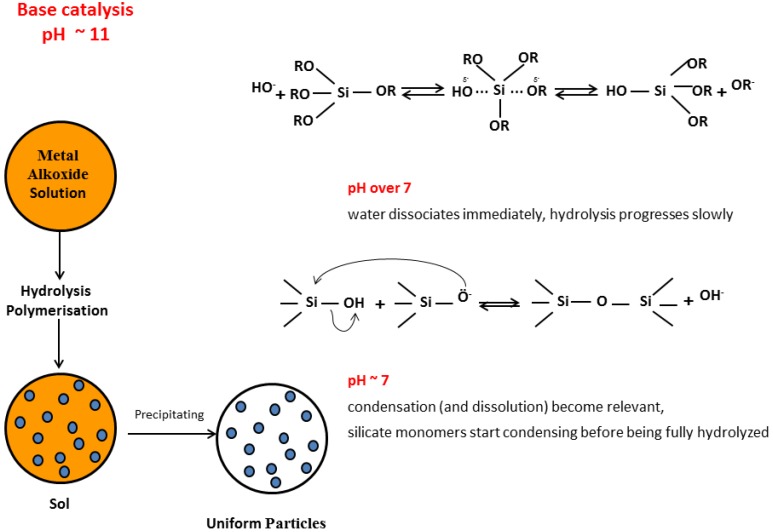
NPs sol-gel Stöber synthesis method.

Sol-gel basic conditions confer negative surface charges to the silica monomers (pH >> pH_PZC_, and ζ ≤ −30 mV), which (kinetically) stabilize the silica suspension, allowing the formation of NPs. Above pH 7, maximum NPs growth is achieved, as a consequence of the increase in silica solubility, which promotes depolymerization of siloxane bonds, and produces monomeric silica necessary for the aging process. As to NPs, Stöber developed a mild synthetic protocol (room temperature, pH ~9–11) for growing (quasi)monodispersed spherical NPs (with diameters between 50 nm and 2 mm) based on sol-gel silicon alkoxides and sodium silicate solution (SSS) as seeds (Figure 8). An alkoxide precursor (such as TEOS) is hydrolyzed (in an ethanol solution) to produce silicic acid, which then undergoes a condensation reaction to form amorphous silica NPs. Arkhireeva and Hay [133] obtained sub-200 nm NPs by slightly modifying the Stöber method. On the other hand, synthesized SiO_2_ NPs (in sub-100 nm size range) present high polydispersity and irregular shape. Zou et al. [30] proposed a procedure to produce monodisperse spherical SiO_2_ NPs with sizes ranging between 30–100 nm, based in the classical two-dimensional LaMer [134] model. The strategy is built upon an effective selection of reaction conditions for the Stöber method, and relies on a modification of the conceptual classical LaMer model of nucleation and particle growth. The LaMer methodology, supported on the protocols by Arkhireeva et al. [133] allow the synthesis of NPs at room temperature in less than 1 h.

Generally acid conditions favor the production of gels, as the silica formed in acid solutions possesses little or no surface charge (zeta potential will be in the tricky range of ζ < |30 eV|, PZC silica ~ pH = 2.2) facilitating flocculation/connectivity between silica species. Here the hydrolysis step is typically the fastest, but condensation begins before hydrolysis is complete. Condensation often occurs in terminal silanols, resulting in chain like structures in the sol and network-like gels. Linear or highly branched polymeric species are formed, given rise to 3D structures.

**Figure 9:**
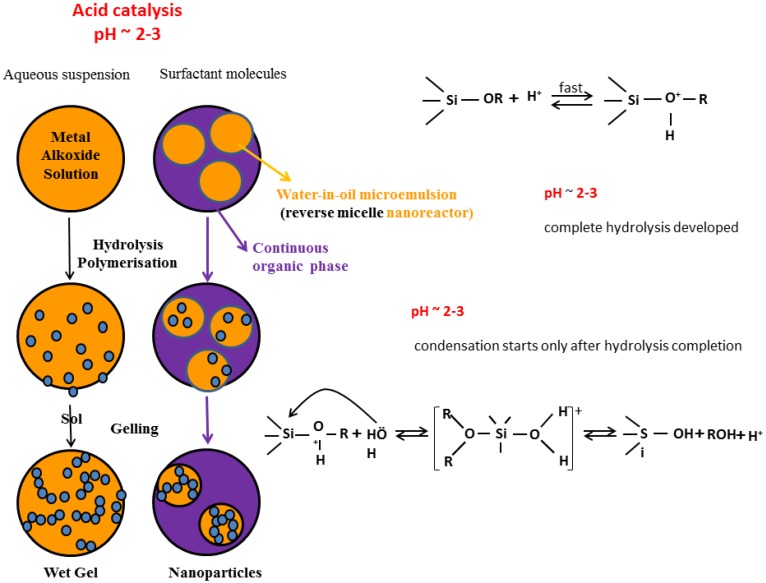
NPs sol-gel reverse emulsion synthesis method.

To synthetize SiO_2_ NPs under acid-catalyzed process a reverse-micelle (or water-in-oil microemulsion) system is formed by adding water, oil and surfactant. The hydrolysis and condensation reactions will develop in the confined reaction vessels (formed by the dispersed aqueous phase in the continuous oil matrix (Figure 9)). The confined nanoreactor environment is shown to yield highly monodisperse NPs and allow the incorporation of non-bonded non-polar molecules, which are often difficult to incorporate into the hydrophilic silica matrix. In the last few years, several dye-doped SiO_2_ NPs have been synthesized by the reverse microemulsion technique in which polar dye molecules are used to ensure successfully encapsulation into SiO_2_ NPs [135].

The reverse microemulsion process is widely used in silica NPs synthesis. However, besides having low yields, the reverse microemulsion process uses a large amount of potentially toxic surfactants and organic solvents, and demands previous washing to biological application, in order to avoid disruption or lyses of biomembranes. The Stöber’s method arises as more eco-friendly alternative, in which the hydrolysis and condensation of a mixture of alkoxysilanes takes place in mild basic aqueous medium, to create monodisperse, spherical, electrostatically-stabilized particles. Recently (ammonia free) Stöber silica NPs were synthesized under basic catalyzed ensured by hydrothermal water (SPA Cabeço de Vide, Portugal, pH ~11) [136].

The Stöber method is a promising method for producing surfactant free silica NPs or coatings; yet the final particles size remain in the hundreds of nanometers to micron regime, which are too large to some of the biological studies. LaMer alternative allows the control of particles size and dispersion, but a regular shape (silica NPs < 100 nm) is still difficult to obtain. NPs prepared through the microemulsion method, exhibited smooth surfaces and low polydispersity. However, for use in biomedicine, the microemulsion method is not as safe as the Stöber one; the use of surfactants in the NPs synthesis carries a higher risk of cytotoxicity.

Stöber silica NPs are largely used in oral applications on account of their chemical stability and intrinsic hydrophilicity, being thus appropriate for biological environments. AEROPERL^®^ 300 Pharma (particle size of 30 mm) is used in formulations of hesperidin oral delivery carrier [137], hydrophylic Aerosil 380 (7 nm in size) is used to stabilize Pickering emulsions in lipid-based oral delivery systems [138,139,140]. Oral insulin bioavailability was tested in a SiO_2_ nanoplatform (silica NPs associated with insulin and then coated with mucoadhesive polymer, like chitosan or PEG) [100,102] (Table 4).

Sol-gel allows in situ incorporation of a variety of functional (non-hydrolysable) organic groups within the silica matrix, in order to increase their biocompatibility, improve its resistance to enzymatic action, internalization efficiency and gene targeting (either in Stöber or reverse emulsion methods). The ORganically MOdified SILica matrix (known as ORMOSIL [141,142]) is an alternative material with even better and more versatile properties than silica: the presence of non-hydrolysable organic groups in the alkoxisilane precursors makes these behave like glass modifiers, reducing the degree of silica network cross-linking as well as increasing the network flexibility as the unhydrolyzed—Si-R bond apparently dangles, causing higher mobility during gelation and undergoing weaker contraction during drying. A tunable wettability, by a judicious choice of the ratio of hydrophilic to hydrophobic sol-gel precursor monomers, a tailor made porosity (size and shape) and a shell hardness/complacency making ORMOSIL a very competitive material. Furthermore, ORMOSIL NPs surfaces will be populated with both silanol and non-hydrolysable organic groups, allowing an easier chemical conjugation/decoration of biomolecules at the NPs surface and/or be loaded with either hydrophilic or hydrophobic drugs or dyes. Mammalian cells take up and internalize easily silica/ORMOSIL NPs (without any cytotoxic effect) opening the door to its use in health science [143].

Among the commonly used functionalizing groups, amine (–NH_2_) is the first choice when gene transfection is designed for gene therapy or vaccination. The –NH_2_ groups electrostatically interact with proteins, enhancing their absorption, biding and protecting pDNA from enzymatic digestion allowing cell transfection in vitro. ORMOSIL NPs have great potential in DNA delivery; ORMOSIL transfection efficiency was equal to or even better than Herpes Simplex Virus-1 (HSV-1)) and does not cause any damage to the tissue nor has immunological side effects that have commonly been observed with viral-mediated gene delivery [144]. ORMOSIL NPs crossed the blood brain barrier (BBB) in fruit fly insects [145] where no toxic effects on the whole insect organism or their neuronal cells were observed. Biodistribution and clearance in vivo studies (mice) using ORMOSIL NPs showed a greater accumulation in liver, spleen and stomach than in kidney, heart and lungs. Although, clearance studies carried out over 15 days period indicated hepatobiliary excretion of the NPs in the same mice [146].

**Figure 10:**
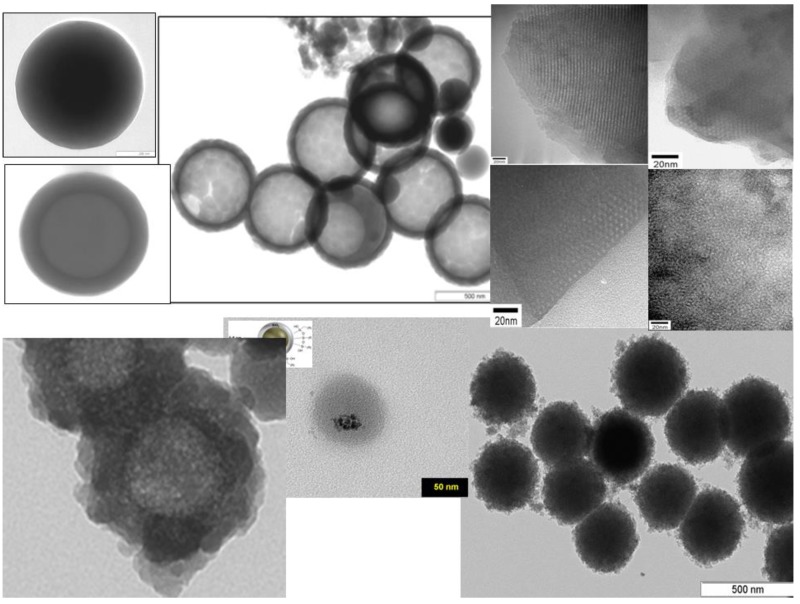
Examples of sol-gel NPs core-shells possibilities (from left-to-right, up-to-bottom): hollow-spheres (core, core-shell and hollow sphere); nanostructure mesoporous spheres; LIPOSIL structure; SPION-core-silica-shell and ORMOSIL NPs.

Core-shell structures have great potential in future biomedical applications, since they constitute a scaffold to create multifunctional NPs, applied to several medical fields, from theranosis to gene delivery performance. Sol-gel Stöber method, by simply replacing the nucleating agent SSS (commonly used in the synthesis of plain SiO_2_ NPs) by another nanosized system, enables its coating. Superparamagnetic iron oxide NPs (SPIONs) [48], and liposomes [147] are selected nanosystems, due to their academic and industrial relevance (Figure 10).

SPIONs, the only clinically approved metal oxide NPs [48], have an excellent response to external magnetic fields. However, administration route and SPIONs surface properties dictate their ultimate effect in terms of the efficiency of cellular uptake, biodistribution, and potential toxicity. SPIONs with hydrophobic surfaces are rapidly and efficiently opsonized and cleared from mammal’s circulation system, while SPIONs with hydrophilic surfaces resist these processes being slowly cleared. Silica/ORMOSIL coating emerge as an interesting coating material, granting hydrophilic surface properties, decreasing SPIONs high aggregation tendency, protecting SPIONs from oxidation and thus increasing their blood circulation time [148].

Liposomes are excellent carriers due to their capacity to load hydrophilic and/or hydrophobic molecules, to penetrate in altered vasculatures (due to pathological situations like in cancer or inflammation), to drug release at target sites (over prolonged periods which may vary from hours to weeks) [149]. In clinic, for intravenous administration, there are already several pharmaceutical systems where drugs are encapsulated in liposomal structure. However, when oral administration is envisaged and gastrointestinal tract mucus and epithelium barrier need to be overcome, the protection of liposomes from anticipated disruption becomes a promising strategy [150]. The emerging of silica-based drug delivery carriers for oral route administration was the *leitmotiv* for silica-coating of liposomes, LIPOSIL for short [151].

Simply silica hollow-sphere NPs (another core-shell possibility) are capable of carrying large amounts of payload or fill their cores with other desirable materials such as polymers, gold or silver along with the gene delivery performance. They can be created through the condensation of alkoxysilanes onto polymer based templates, metal organic frameworks or other nanomaterials, lately removed by chemical etching or thermal degradation [150].

#### 3.3.4. Template Syntheses

**Figure 11:**
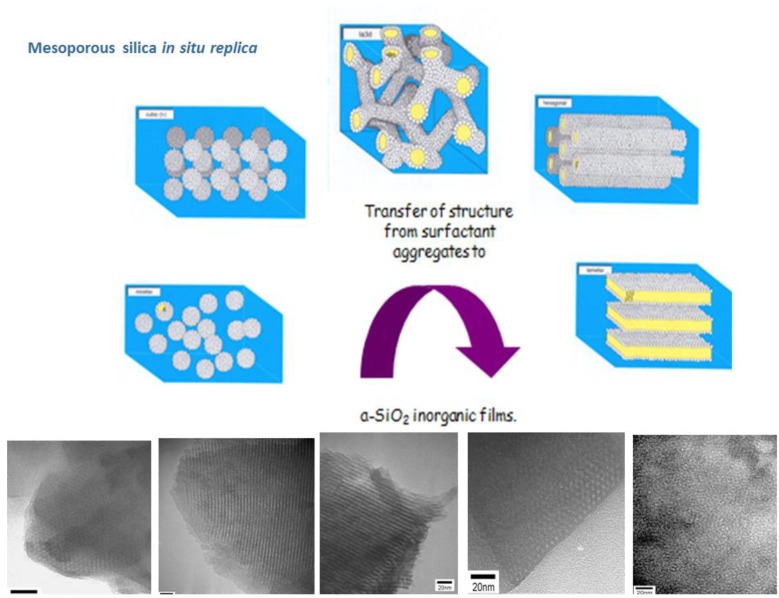
Sol-gel mesoporous silica NPs from in situ *replica* of self-assembled molecular aggregates.

Template synthesis is dedicated to the production of mesoporous materials, topic out of the scope of this short review. A very short summary of synthesis process is presented. The seminal work conducted by researchers at the Mobil Oil Corporation in the early 1990s on the synthesis of mesoporous silicates has led to a number of syntheses in which surfactants are used as templates [152]. Ordered mesoporous materials are unique materials that are defined by an ordered and repetitive mesostructured of pores and disordered arrangement at the atomic level. Their synthesis is based on the use of surfactants that act as templates to direct the morphology of the final amorphous material. Simply, the synthesis process starts with the dissolution of surfactant molecules into polar solvents to yield liquid crystal suspensions. The pair surfactant/solvent defines the working phase diagram. When the surfactant concentration is above the critical micellar concentration (CMC) then the surfactant molecules self-assembly into micelles. Higher surfactant concentrations allow the formation of micellar cubic, hexagonal or lamellar self-assembly structures. Once the (liquid crystal) aggregates are formed, the silica precursors are added to the suspension, the sol-gel reactions occurs and a mesoporous silica material is produced. Finally, the surfactant is removed by chemical or thermal degradation [29] (Figure 11).

A definite breakthrough in drug delivery was the use of mesoporous silica NPs to host drugs/therapeutic-molecules/markers. A correct selection of the mesoporous design depends on the molecule to be hosted, and is the first criterion used. The most used mesoporous silica NPs in drug or bioencapsulation are MCM-41 and SBA-15. The synthesis of MCM-41 (from the Mobil Composition of Matter series) involves liquid crystal templating commonly cetyl trimethylammonium bromide (CTAB) that lead to a 2D hexagonal pore channel array with 3.6 nm in size. The diameters of MCM-41 NPs can be controlled in a size range from 25 nm to 100–150 nm. The SBA-15 (Santa Barbara type) is also largely used as biocarrier. This type of mesoporous silica material is prepared by cooperative self-assembly with a pluronic P123 (a non-ionic block co-polymer). The channels adopt also a 2D hexagonal packing with a diameter varying from 6 to 10 nm depending on the synthesis conditions.

The release of the drug from the host mesoporous NP is definitely the big challenge. This may occur through diffusion all through the pore channels (in passive drug delivery) or released under specific stimuli as pH, temperature, ultra-sons or light (in stimuli-responsive systems). Although an impressive variety of mesoporous NPs have design, synthesized and (in vitro and in vivo) tested no products have reached the market so far.

## 4. Conclusions

Silica, the major component of the Earth’s crust, has entered the food chain and become an essential material in many, if not all, forms of life. Although in humans its role in disease, aging and health is not yet fully understood, its simply presence in the body renders it a biocompatible material. In nanomedicine silica has become an excellent competitor as a nanocarrier, able to target, sense, signal, and drug release drugs/markers with different pharmacokinetics/pharmacodynamics, to elaborate optimized tailor made treatments/diagnosis.

Currently pharmaceutical design is based on Quality by Design concept, which demands accurate knowledge of the physiological barriers nanocarriers face within the human body, of the physical/chemical characteristic of nanocarriers besides fully understand of their synthesis process. A comprehensive revision about these topics is present. In particular, the sol-gel silica nanoparticles synthesis, namely colloidal route, biomimetic production, solution route and template synthesis are discussed, having the ecological inference in consideration. The Stöber and LaMer solution route protocols stand out as time-saving and surfactant free processes. Mimetic natural silica production (either geologic or biologic processes) is gaining ground, and is an inspiration of green eco-production processes. A considerable number of commercial, approved, patented or in clinical stage sol-gel silica NPs are presented. Taken together, the examples shown in this short review emphasize the great potential of silica nanoparticles as nanocarriers in medicine. Improve the synthesis process and design more competitive NPs to comply the rising human health demands are the challenges chemists and materials scientist will face in the near future.

## Figures and Tables

**Table 1:** Multifunctional NPs: materials and functions (adapted and reproduced with permission from [14]).

Component	Material	Function
Biomedical payload	Imaging agents for optical, MRI, MPI, CT, PET, SPECT, US imaging (organic dye, QDs, UCNPs, magnetic materials, metal NPs)	Image enhancement
	Therapeutic agents (anticancer drugs, DNA, siRNA, hyperthermal/photodynamic materials)	Cancer cell death induction, gene up/down regulation
Carrier	Organic (lipid, natural/synthetic polymers)	Multifunctional (protection of payloads, controlled release of drug/gene, biocompatibility, stimuli responsiveness)
	Inorganic (hollow metal NPs, hollow metal oxide NPs, C nanostructures, porous, non-porous, core-shell or nanostructured SiO_2_ NPs)	Multifunctional (imaging ability added to above functions)
Surface modifier	Antibody	Molecular imaging
	Aptamer	Target specific delivery
	Peptid/protein	Uptake enhancement
	Small molecules	Penetration of barrier
	Charge balancing molecules	Signaling transduction Stimuli responsiveness

**Table 2:** Sol-gel silica nanocarriers in clinical use and under clinical investigation.

Trade Mark	Formulation	Company	Application	Phase of Development
C-dots^®^	PEG-coated SiO_2_ NPs	C-dots Development (USA)	Melanoma (Intravenous)	FDA approved 2011
PreveCeutical^®^	SiO_2_ sol-gel delivery platform	PreveCeutical (Canada)	platform for Nose-to-Brain Delivery of Therapeutic Compounds	INC FDA approval
Vered^®^	Patented microencapsulation SiO_2_ NPs	Sol Gel Technologies (Israel)	Papulopostular Rosacea (Dermatology)	Phase II
Twin^®^	Patented microencapsulation SiO_2_ NPs	Sol Gel Technologies (Israel)	Acne Vulgaris (Dermatology)	Phase II
Sirs-T^®^	Patented microencapsulation SiO_2_ NPs	Sol Gel Technologies (Israel)	Acne Vulgaris (Dermatology)	Phase II
Generic	Patented microencapsulation SiO_2_ NPs	Sol Gel Technologies (Israel)	Acne Vulgaris (Dermatology)	Phase III
	Ultra-small silica-based bismuth gadolinium NPs	NH TherAguix (France)	Dual MR-CT guided radiation therapy	Phase I
AbsolutMag™	Silica NPs, TiO_2_-SiO_2_ coated NPs (10, 20, 30 nm, 20 µm)	Cd Creative Diagnostics (USA)	Theranostic	Phase I
DiagNano™	Silica magnetic NPs (produced by hydrolysis of orthosilicates in the presence of magnetite) (250 nm–6 µm; 6.0–43 emu/g)	Cd Creative Diagnostics (USA)	DNA/RNA isolationa and purification	Phase I
AuroLase^®^	PEG-coated silica-gold nanoshells	NanoSpectra Biosciences (USA)	Near-IR light facilitated thermal ablation. Thermal ablation of solid primary and/or metastatic lung tumors	NCT01679470 (Not Provided)
AuroLase^®^	PEG-coated silica-gold nanoshells	NanoSpectra Biosciences (USA)	MR/US Near-IR light facilitated Prostate Gland Tumors thermal ablation.	Phase II

**Table 3:** Sol-gel silica-based NPs therapeutics recently patented (based on [49]).

Patent	Title	Inventors
PT20131000062306 PCT/PT2014/000054	Multifunctional Superparamagnetic Nanosystem as Contrast Agent for Magnetic Resonance Imaging and Its Production Method	Gonçalve M.C.; Fortes L.M.; Martins B.M.; Carvalho A.D.; Feio G.
WO2011003109, 2011	Fluorescent silica-based NPs	Bradbury M.; Wiesner U.; Penate M.O.; Ow H.; Burns A.; Lewis J.
US20100055167 Al, 2010	Stem cell delivery of antineoplasic medicine	Zhang A.; Guan Y.; Chen L.
US20107799303, 2010	Method of preparing silica NPs from siliceous mudstone	Jang H.-D.; Chang H.-K.; Yoon H.-S.
US20100303716 Al., 2010	Switchable nano-vehicle delivery systems, and methods for making them.	Jin S.; Oh S.; Brammer K.; Kong S.
WO2009064964, 2009	Switchable nano-vehicle delivery systems, and methods for making and using them.	Jin S.; Oh S.; Brammer K.; Kong S.
US20110092390, 2010	Methods for making particles having long spin-lattice relaxation times.	Marcus C.M.
US20100040693, 2010 WO2008018716, 2008	Silica capsules having nano-holes or nano-pores on their surfaces and method for preparing the same	Chung B.H.; Lim Y.T.; Kim J.K.
US20100255103, 2010	Mesoporous silica NPs for biomedical applications	Liong M.; Lu J.; Tamanoi F.; Zink J.I.; Nel A.
US20100104650, 2010	Charged mesoporous silica naoparticles-based drug delivery system for controlled release and enhanced bioavailability	Lee C.-H.; Lo L.-W.; Yang C.-S., Mou C.-Y.
US201001361124, 2010 WO2008128292, 2008	Nanoparticle-coated capsule formulation for dermal drug delivery	Prestidge C.A.; Simovic S.; Eskandar N.G.
US20090263486, 2009	Nanoparticle-stabilized capsule formulation for treatment of inflamation	Prestidge C.A.; Simovic S.
US20090181076, 2009	Drug release from nanoparticle-coated capsules	Prestidge C.A.; Simovic S.; Eskandar N.G.
WO2009021286, 2009	Organosilica encapsulated NPs	Qiao S.; Lu G.Q.
WO2009091992, 2009	Repairing damaged nervous system tissue with NPs	Cho Y.; Shi R.; Ivanisevic A.; Borgens R.
US20090169482, 2009	Silica-cored carrier nanoparticle	Zhen S.; Dai L.; Wang R.; Qiao T.A.; Che W.; Harrison W.J.
US20090232899, 2009 WO2005117844, 2005	Mucoadhesive nanocomposite delivery systems	David A.E.; Zhang R.; Park Y.J.; Yang A.J.-M.; Yang V.C.
US20090252811,2009 WO2005009602,2005	Capped mesoporous silicates	Lin V.S.-Y.; Lai C.-Y.; Jeftinija S.; Jeftinija D.M.
EP 20070829819, 2007	Mesoporous silica particles.	Yano T.; Sawada T.
WO2005044224, 2005	Drug Delivery system based on polymer nanosshells.	Gao J.; Al H.
GB2409160(A), 2005 WO2004GB05203,2005	A method of engineering particles for use in the delivery of drugs via inhalation	Okpala J.

**Table 4:** Sol-gel silica-based oral delivery NPs (adapted and reproduced with permission from [102]).

Oral Delivery System	Silica Source	Payload	Coating	Encapsulation Method	Release Mechanism	In Vitro/In Vivo/Ex Vivo	Ref.
**Non-porous SiO_2_ NPs**							
Stober NPs	TEOS	Insulin	PEG 6000PEG 20,000	Physisorption of insulin to as-synthesized SiO_2_ NPs–subsequent PEG coating	Passive diffusion	Ex vivo permeation studies with everted rat intestine	[103]
Stober NPs	TEOS	Insulin	Chitosan	Physisorption of insulin in chitosan suspension to as-synthesized SiO_2_ NPs	Passive diffusion	In vitro studies of NPs interactions with porcine mucin	[104]
**Mesoporous SiO_2_ NPs**							
MCM-48	Luox AS40	Ibuprofen		Physisorption by immersion	Passive diffusion	In vitro drug release in a simulated body fluid (pH 7.4–7.7)	[105]
Ia3d MSM	TEOS/MPTS	Erythromycin					
SBA-15 SiO_2_	nf	Itraconazole		Physisorption by immersion	Passive diffusion	In vitro drug release in a simulated gastric fluid (pH 1.2)	[106]
SBA-15 and MCM-41 functionalized with –NH_2_ groups	nf		Bisphosphonates	Electrostatic interaction between drug’s phosphate group and silica’s amnine group at pH 4.8	Passive diffusion t pH 7.4	In vitro drug release in phosphate buffer (pH 7.4)	[107]
MCM41 microparticles	TEOS/tri-ethanolamine	Folic acid		Impregnation	pH triggered	Yoghurt in vitro drug release in a simulated GIT fluid (pH 2, 4, 7.5)	[108]
MCM41 NPs	nf	Rhodamine B	a-CD, adamantly ester	Physisorption	Porcine liver esterase triggered	In vitro hydrolysis in HEPES buffer pH 7.5	[109]
MCM48	TEOS/APTES	Silfalazine	Succinylated soy protein isolate	Physisorption and coating	pH/enzyme triggered	In vitro drug release in simulated GIT fluid at pH 1.2, 5, 7.4	[110]
**Hybrid silica microparticles**							
Core-shell (mesostructured SiO_2_)	TMOS	Curcumin		1. Encapsulation of curcumin in SLN by emulsification/sonication 2. sol-gel	Passive diffusion	In vitro drug release in a simulated GIT fluid (pH 1.2–7.4)	[111]
Core-shell alginate SiO_2_	TMOS/APTMS	LGG		1. Preparation of LGG/alginate microgels by electrospraying 2. mineralization	Erosion of silica shell	In vitro drug release in a simulated GIT fluid (pH 1.2–7.4)	[111]
**Diatoms silica microparticles**							
Diatom silica	fossile	Indomethacin/gentamicin		Physisorption	Passive diffusion	In vitro drug release in a simulated intestinal fluid (pH 7.2)	[112]
Diatom silica	fossile	Mesalamine/prednisone		Physisorption	Passive diffusion	In vitro drug release in a simulated GIT fluid (pH 1.2–7.4)	[97]
